# Vector- and Rodent-borne Diseases in Europe and North America: Distribution, Public Health Burden and Control

**DOI:** 10.3201/eid1308.070626

**Published:** 2007-08

**Authors:** Alan D.T. Barrett

**Affiliations:** * University of Texas Medical Branch at Galveston, Texas, USA

**Keywords:** vectorborne diseases, rodentborne diseases, Europe, North America, book review

An estimated 70% or more of emerging infectious disease agents have some form of vector. The author of this book, Norman Gratz, a medical entomologist, has written a very valuable resource on vectorborne and rodentborne diseases found in Europe and North America. His book includes information on diseases transmitted by mosquitoes, ticks, rodents, mites, sandflies, fleas, lice, biting midges, diptera, triatomines, and cockroaches. As a text, it is impressive that one man has such a breadth of knowledge of these diseases, although some credit must go to Mike Service, who helped prepare the manuscript for publication after the death of the author.

Unlike most books on this subject, which concentrate on describing the agent and its molecular properties, this book focuses on public health aspects of the diseases, and most chapters are divided on the basis of the vector that carries the agents. The chapters describe the history of the agents and details of incidence by country, year, and important ecologic parameters. The details of the number of cases of each disease by year are particularly impressive. Many chapters have a conclusion section with an overview of the public health importance of the disease, interpretation of the risk of the disease, and identification of our knowledge gaps. In addition, some chapters are devoted to the economic effects of vectorborne and rodentborne diseases in Europe and North America.

This is a very readable text or reference book for those who want to know about a specific vectorborne or rodentborne disease in Europe or North America through 2003–2004. The only weakness I could find is that although the word “control” is used in the title, the book contains relatively little information on this subject. However, the depth of other areas compensates for a lack of information about control.

Overall, I recommend this to anyone who needs a reference book on vectorborne and rodentborne diseases. All we need now is an equivalent reference for such diseases in Africa, Asia, Australasia, and South America!

